# Enhancing HIV-1-specific CAR-T cell functional activity by treatment with a novel cytokine-based scaffold with IL-15 superagonist and TGF-β-neutralizing activity

**DOI:** 10.1128/jvi.00051-26

**Published:** 2026-08-14

**Authors:** Sara Lamcaj, Erin Cole, Christopher Hiner, Marta Santos Bravo, Jian Hua Zheng, Niraj Shrestha, Gregory Sowd, Ying Xiong, Zhongyu Zhu, Boro Dropulic, Hing C. Wong, Harris Goldstein

**Affiliations:** 1 Department of Microbiology and Immunology, Albert Einstein College of Medicine 2006, Bronx, New York, USA; 2 HCW Biologics Inc., Miramar, Florida, USA; 3 Caring Cross, Gaithersburg, Maryland, USA; 4 Department of Pediatrics, Albert Einstein College of Medicine 2006, Bronx, New York, USA; Icahn School of Medicine at Mount Sinai, New York, New York, USA

**Keywords:** TGF-β, immunotherapy, HIV-1, CAR-T cells, DuoCAR-T cells, IL-15, HIV cure strategy, immune suppression, immune modulation, gp120/gp41, p24, capsid

## Abstract

Chimeric antigen receptor T cell (CAR-T cell) therapy targeting and eliminating HIV-infected cells offers a promising approach to provide people living with HIV (PLWH) with a functional cure by preventing the recurrence of viremia caused by reactivation of latent HIV-1-infected cells. We previously described a bispecific CAR-T cell targeting two highly conserved gp120 epitopes (duoCAR-T cell) with potent anti-HIV-1 activity that is currently in clinical trials. However, elevated levels of transforming growth factor β (TGF-β) present in many PLWH may hinder the activity of both infused HIV-1-specific CAR-T cells, such as duoCAR-T cells and endogenous HIV-1-specific CD8^+^ T cells, thereby limiting their effectiveness to achieve a functional HIV-1 cure. We hypothesized that HCW9218, a novel bifunctional immunomodulatory protein composed of TGF-βRII and IL-15/IL-15Rα, would enhance anti-HIV-1 immunity by TGF-βRII binding and neutralizing TGF-β, while IL-15/IL-15Rα would stimulate effector cells and reactivate latent HIV-1-infected cells. We used duoCAR-T cells generated from CD4^+^ and CD8^+^ T cells from people without HIV (PWoH) and PLWH donors to demonstrate the *in vitro* capacity of HCW9218 to block TGF-β activity, and enhance duoCAR-T cell proliferation, cytotoxicity, and anti-HIV-1 activity. HCW9218 also functioned as a latency-reversing agent, stimulating HIV-1 production by CD4^+^ T cells from ART-suppressed PLWH. Production of HIV-1 by HCW9218-treated CD4^+^ T cells from ART-suppressed PLWH donors was suppressed by co-culture with autologous duoCAR-T cells. Together, these findings highlight the potential of HCW9218 to augment T cell and CAR-T-based therapies and contribute to strategies aimed at achieving a functional cure for HIV-1.

**IMPORTANCE:**

The persistence of HIV-1 reservoirs remains the primary barrier to an HIV-1 cure because antiretroviral therapy (ART) suppresses viral replication but does not eliminate latent HIV-1-infected cells. Treatment with anti-HIV-1 duoCAR-T cells is a potential strategy to target and eliminate HIV-1-infected cells, but their activity may be impaired by the immunosuppressive environment in lymphoid tissues of people living with HIV (PLWH). Transforming growth factor β (TGF-β), a pleiotropic cytokine elevated in PLWH, is a key mediator of this immunosuppression. Here, we show that HCW9218, a bifunctional fusion protein with TGF-β-neutralizing activity and IL-15 superagonist activity, preserves duoCAR-T cell function in the presence of TGF-β and reactivates HIV-1 production by latent HIV-1-infected cells in ART-suppressed CD4^+^ T cells from PLWH. These findings highlight HCW9218 as a unique dual-function immunotherapy that may enhance the efficacy of duoCAR-T cells while facilitating clearance of the HIV reservoir.

## INTRODUCTION

Human immunodeficiency virus type 1 (HIV-1) infection continues to be a significant global health challenge with an estimated total of almost 40 million people living with HIV (PLWH) in 2023. While antiretroviral therapy (ART) suppresses HIV-1 replication, it does not eliminate HIV-1-infected cells, necessitating lifelong treatment and leaving individuals at risk for immune dysfunction and comorbidities. A small fraction of individuals, known as “elite controllers,” can suppress HIV-1 replication without ART, and some studies have linked this control to robust proliferation and cytolytic effector function of their HIV-1-specific CD8^+^ T cells (1–5). During chronic HIV-1 infection, persistent antigen exposure and an immunosuppressive environment can compromise the function of HIV-1-specific CD8^+^ T cells (6–8). In particular, elevated TGF-β production further suppresses immune function and promotes tissue fibrosis, thereby driving disease progression and associated comorbidities (9–11).

TGF-β is a pleiotropic cytokine that regulates immune homeostasis by controlling cell differentiation, activation, and survival, while promoting the development of regulatory T cells (Tregs) that suppress immune activation (12, 13). In many PLWH, TGF-β production is elevated, which has been linked to impaired cellular immune responses (14–16). Higher TGF-β levels correlate with disease progression primarily in settings of untreated HIV-1 infection, and studies have shown that blocking TGF-β during uncontrolled infection or analytical treatment interruption can reactivate latent HIV-1 and SIV reservoirs and restore HIV-1/SIV immune responses *in vivo* (17–19). These findings indicate that TGF-β inhibition might be used as an adjunctive therapeutic strategy for treating HIV-1 and providing a functional cure in combination with other immune-based interventions.

Immunotherapeutic strategies, including cytokine therapy, immune checkpoint inhibitors, and cellular therapies such as CAR-T cells, have revolutionized oncology treatment by amplifying immune activity to overcome limitations in endogenous responses (20–22). Combination therapies that pair immunostimulants, such as IL-15 or IL-7, with simultaneous blockade of inhibitory signals synergize to enhance immune responses (23–25). IL-15, produced by mononuclear phagocytes, T cells, and dendritic cells, is critical for the development, proliferation, and activation of T cells and natural killer (NK) cells (26, 27). IL-15 also supports the generation and maintenance of memory CD8^+^ T cells (28) and sustains antigen-specific effector CD8^+^ T cells during the contraction phase (29). These properties make IL-15 an attractive candidate for boosting both antiviral immunity and antitumor responses in the treatment of viral infections and cancer, respectively.

IL-15 activity can be further boosted and prolonged by associating IL-15 with the IL-15Rα subunit to generate an IL-15 superagonist that directly trans-presents IL-15 to target T cells and NK cells (30–32). The IL-15 superagonist, N-803 (Anktiva), received FDA approval in 2024 for treating some bladder cancers. In preclinical studies using SIV-infected rhesus macaques, N-803 administration promoted SIV-dependent CD8^+^ T cell and NK cell proliferation associated with reductions in plasma viral loads (33–37). Furthermore, early studies investigating latency reversal demonstrated that IL-15 stimulation increases viral p24 antigen production in the supernatant of latently infected cells, while N-803 induces HIV-1 transcription *ex vivo* from ART-suppressed donors (38). These findings underscore the therapeutic potential of IL-15 superagonists to boost HIV-1-specific cellular immunity in parallel with reactivating latent HIV-1-infected cells.

We previously developed and validated an anti-HIV-1 duoCAR-T cell therapy that potently suppressed HIV-1 infection *in vitro* and *in vivo* in a humanized mouse model of HIV-1 infection (39, 40). The duoCAR targets two conserved HIV-1 gp120 Env glycoprotein epitopes involved in HIV-1 binding to CD4 (md1.22) and the coreceptor (m36.4) (39). Studies using humanized mice demonstrated that the anti-HIV-1 duoCAR-T cells migrated to active HIV-1 infection sites within the humanized mouse spleens, where they suppressed HIV-1 infection (40). As TGF-β is elevated in PLWH and known to impair T cell function, we hypothesized that combining IL-15 stimulation with TGF-β blockade would enhance the anti-HIV-1 activity of duoCAR-T cells. In this study, we demonstrate that a bifunctional molecule that combines IL-15 with a TGF-β inhibitor, termed HCW9218 (23), can counteract the immunosuppressive effects of TGF-β while stimulating the proliferation and cytotoxic function of anti-HIV-1 duoCAR-T cells. HCW9218 is a two-chain protein encoded by separate constructs, with one chain consisting of a dimerized human TGF-β receptor II extracellular domain (TGF-βRII) linked to the extracellular domain of tissue factor (TF), which is also fused to a human IL-15 molecule. The second chain contains a dimerized TGF-βRII domain linked to the extracellular human sushi domain of human IL-15Rα (IL-15RαSu). When these proteins are mixed, binding of the IL-15 domain on one chain to the IL-15RαSu domain on the second chain forms the HCW9218 heterodimer. The TGF-βRII domains of this protein bind to and neutralize TGF-βI, while the IL-15RαSu domain functions as an IL-15 superagonist. Our findings in the current study describe a novel therapeutic strategy for HIV-1 treatment that leverages the synergistic effects of IL-15 immune stimulation and TGF-β neutralization to enhance the anti-HIV-1 activity of duoCAR-T cell therapy.

## RESULTS

### HCW protein constructs

**Fig 1:**
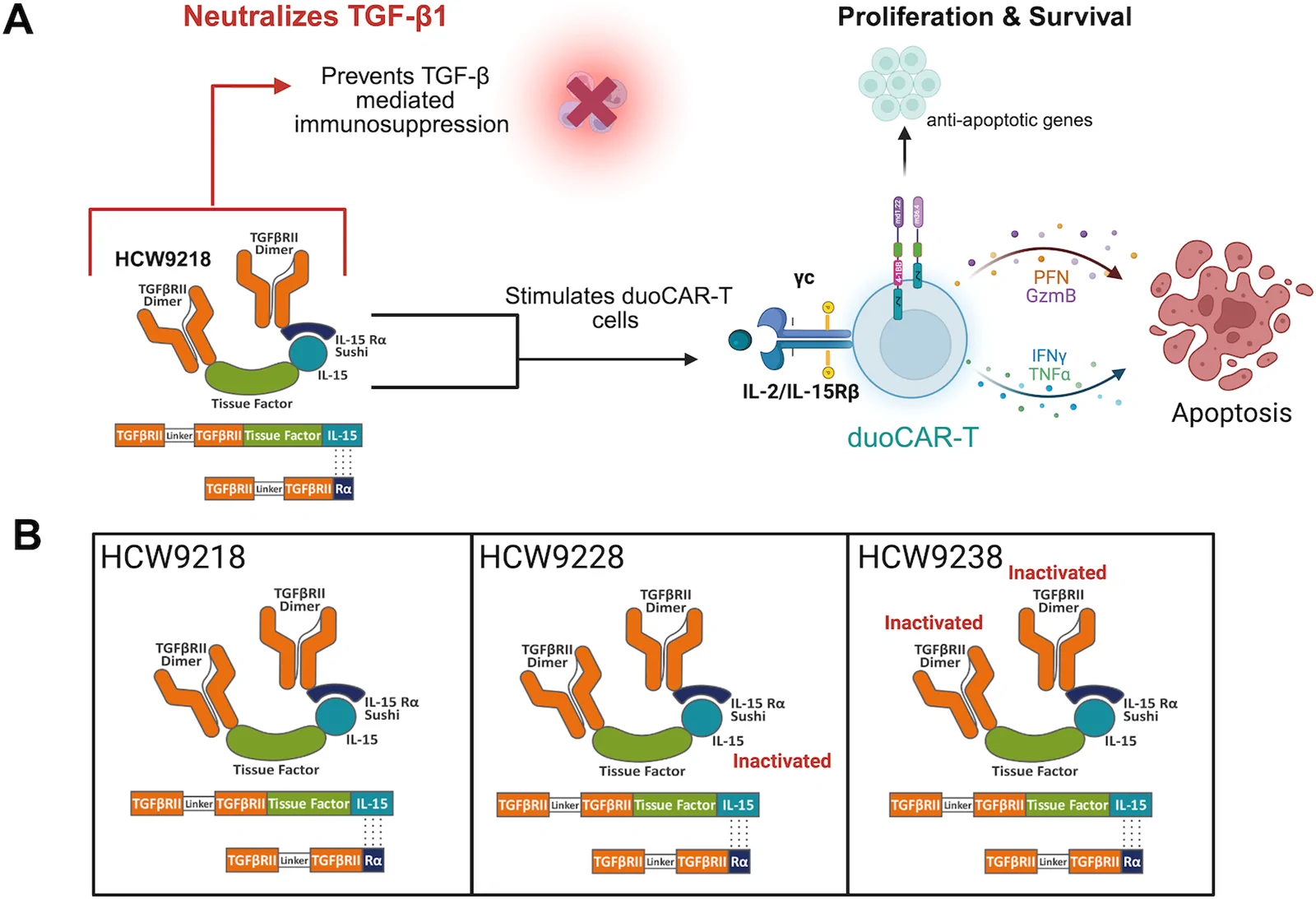
Schematic overview of HCW proteins. (**A**) Protein schematic of the structure and function of the heterodimeric bifunctional fusion protein complex, HCW9218, comprising a tissue factor scaffold linking a TGF-βRII molecule and an IL-15 molecule, which binds the IL-15Rα sushi domain linked to a second TGF-βRII molecule. (**B**) Protein schematic of HCW9218, HCW9228, and HCW9238. HCW9228 only has TGF-β binding activity because it contains an IL-15 D8N mutation to abrogate IL-15 activity. HCW9238 only has IL-15 superagonist activity because its TGF-βRII domain is mutated to abrogate its capacity to bind TGF-β.

We previously described HCW9218, a bifunctional TGF-β trap/IL-15 protein complex that we developed to stimulate anti-tumor immune responses, which links an immunostimulatory human IL-15/IL-15 receptor α (IL-15Rα) complex with the extracellular domains of the human transforming growth factor-β (TGF-β) receptor to a tissue factor scaffold that delivers the IL-15 signal and inhibits TGF-β suppression to potently expand T cells (23). In contrast to free IL-2, which can directly bind the complete IL-2Rαβγc complex and stimulate target cells that bind weakly to IL-2Rα alone, free IL-15 binds strongly to the IL-15Rα subunit. IL-15 at lower concentrations has minimal activity unless it is bound to IL-15Rα to enable trans-presentation to the IL-15Rβγc complex on target cells (27, 41). Consequently, we constructed HCW9218 with the human IL-15/IL-15Rα complex that functions as an IL-15 superagonist to provide more potent and sustained activity than IL-15 alone to stimulate CD8^+^ T cells and NK cells and promote their proliferation, activation, function, and survival (27, 41). We postulated that HCW9218 treatment would increase the anti-HIV-1 activity of duoCAR-T cells by inhibiting TGF-β-mediated suppression of duoCAR-T cell function and stimulating duoCAR-T cell proliferation and function (Fig. 1A). To delineate the activity of the TGF-β receptor domain and the IL-15Rα complex, we constructed two additional HCW proteins as controls. The activity of the IL-15 in HCW9228 was attenuated by a D8N mutation that reduces its binding to IL-15Rβ while maintaining its capacity to neutralize TGF-β (42), whereas the TGF-β domain in HCW9238 was mutated to abrogate TGF-β neutralization while maintaining IL-15 superagonist activity (Fig. 1B).

### HCW9218 displays IL-15 activity and neutralizes TGF-β activity

**Fig 2:**
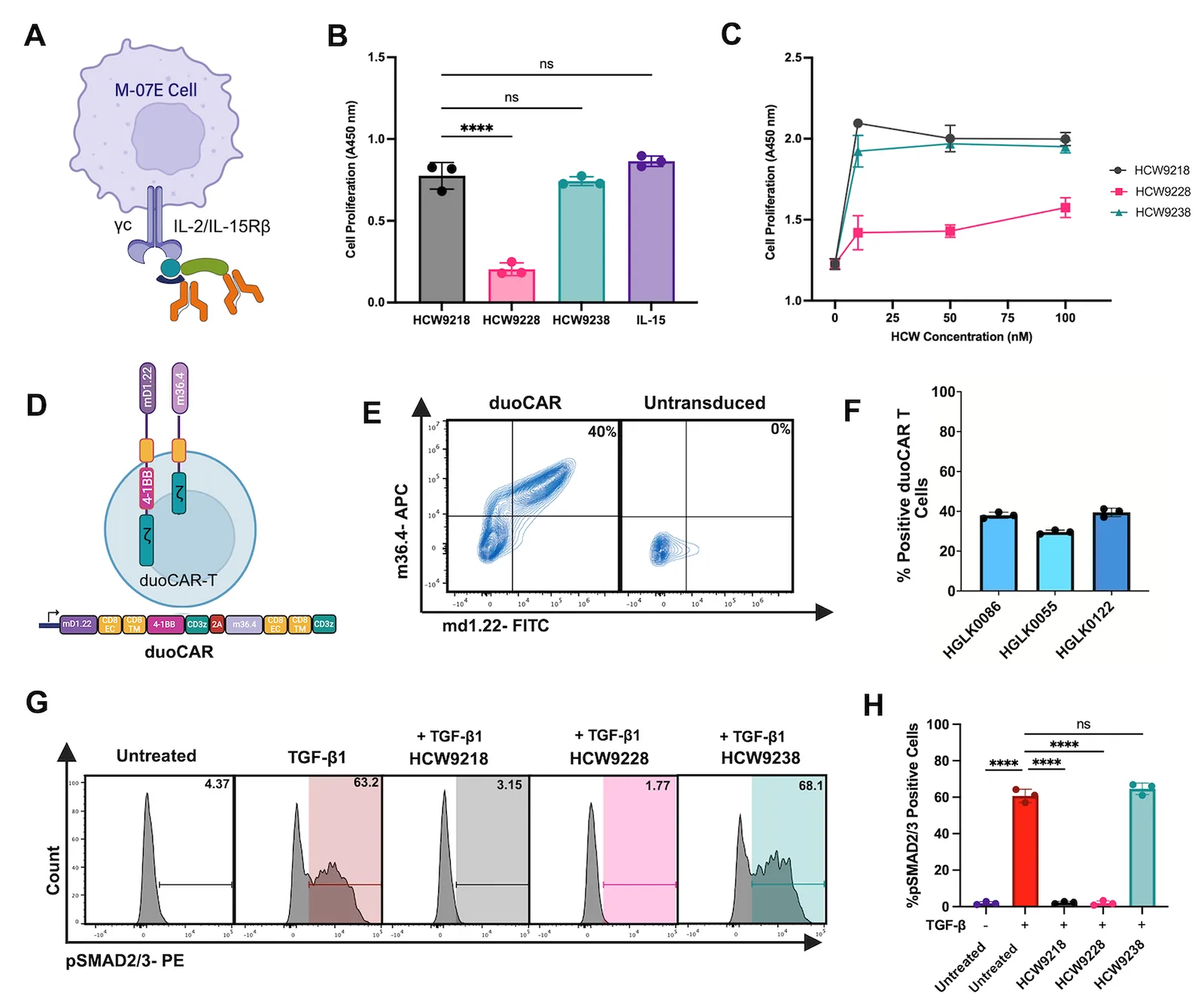
HCW9218 promotes proliferation in an IL-15-dependent cell line and neutralizes TGF-β signaling in duoCAR-T cells. (**A**) Schematic of IL-15-mediated activation of M-07E cells by HCW9218. (**B**) M-07E cell proliferation mediated by 50 nM HCW proteins or 1 nM IL-15 treatment after 3 days as measured using CCK-8 solution with level of absorbance (450 nm) being proportional to cell counts. Data represent absorbance after subtraction of the unstimulated background to show net proliferation and presented as mean ± SD of technical triplicates from one representative experiment out of three independent experiments. (**C**) Dose-dependent proliferation of M-07E cells in response to HCW proteins. Data are presented as mean ± SD of technical triplicates from one representative experiment out of three independent experiments. (**D**) Schematic diagram of duoCAR. (**E**) Representative flow cytometry analysis for CAR expression on engineered primary human T cells. CD4^+^ and CD8^+^ T cells were isolated from PWoH donor PBMCs and were activated with αCD3 and αCD28 for 2 days followed by duoCAR lentiviral vector transduction on day 3. Three days later, T cells were stained to detect duoCAR expression. Cells were gated on lymphocytes > single cells > CD8^+^ with gates set using single-stained compensation. (**F**) Expression of % positive duoCAR T cells generated by transducing T cells from three PWoH donors detected by flow cytometry after gating on lymphocytes > single cells > CD8^+^ > m36.4 and md1.22. (**G**) Representative flow cytometric histograms of pSMAD2/3 expression by duoCAR-T cells after rhTGF-β (10 ng/mL) treatment alone and in the presence of the indicated HCW protein. (**H**) Average percentage of SMAD2/3 positive duoCAR-T cells from three different PWoH donors. Significance for panel B was determined by ordinary one-way ANOVA followed by Tukey’s multiple comparisons test. Significance for panel H was determined by ordinary one-way ANOVA followed by Dunnett’s multiple comparisons test. (*P < 0.05, **P < 0.01, ***P < 0.001, ****P < 0.0001).

We evaluated the functional activity of the IL-15/IL-15Rα sushi domain of HCW9218 by testing its ability to support the proliferation of the IL-15-responsive M-07E cell line (43) (Fig. 2A). M-07E cells were cultured in RPMI media with added fetal bovine serum (FBS) (20% vol/vol), penicillin (100 U/mL), and streptomycin (0.1 mg/mL), and either untreated or treated with HCW9218, HCW9228, or HCW9238 proteins (50 nM), or IL-15 (1 nM). After three days, IL-15-driven cell proliferation was measured using the Cell Counting Kit-8 (CCK-8), which quantifies viable cells based on formazan production measured by absorbance at 450 nm. M-07E cells treated with HCW9218 and HCW9238 displayed significantly increased proliferation compared with M-07E cells either unstimulated or treated with HCW9228 (*P* < 0.0001), which was comparable to the level of proliferation induced by IL-15 (Fig. 2B). Cells treated with HCW9218 and HCW9238 exhibited increased proliferation across all tested concentrations (10 nM, 50 nM, 100 nM) compared with HCW9228 treatment (Fig. 2C). Additionally, TGF-β (10 ng/mL) significantly inhibited the proliferation of M-07E cells treated with either HCW9238 or IL-15 (*P* < 0.0001). In contrast, TGF-β did not suppress the proliferation of M-07E cells treated with HCW9218, demonstrating effective TGF-β neutralization by HCW9218 (Fig. S1). We further evaluated the functional activity of the HCW9218 IL-15/IL-15Rα sushi domain by determining its capacity to phosphorylate and activate STAT5, which is activated by trans-presentation by cells expressing complexed IL-15Rα and IL-15 on their surface to target cells expressing the γc and IL-2Rβ receptor (44). We demonstrated that treatment with HCW9218 stimulated phosphorylation of STAT5 in donor T cells by flow cytometric analysis of T cells stained with antibodies to phosphorylated STAT5 (Fig. S2).

We further evaluated the capacity of the TGF-βRII domains in HCW9218 to inhibit TGF-β signaling by determining their capacity to block TGF-β-mediated activation of receptor-associated serine/threonine kinases, which stimulate the phosphorylation of SMAD2 and SMAD3 (12, 45, 46). These phosphorylated SMAD proteins (pSMAD2/3) form a complex with SMAD4, translocate to the nucleus, and mediate the functional activity of TGF-β by modulating gene transcription (12, 13). As target cells, we used duoCAR-T cells, which express an anti-HIV-1 duoCAR construct we developed (39) composed of two HIV-1-targeting domains: mD1.22, an engineered single-domain CD4 that targets the conserved CD4 binding site on HIV-1 gp120, and m36.4, a broadly neutralizing heavy-chain-only antibody that binds to the conserved CD4-induced co-receptor binding site on gp120 (Fig. 2D). T cells transduced with the duoCAR vector express comparable levels of both CAR molecules, as demonstrated by flow cytometry (Fig. 2E). We observed consistent production of duoCAR-T cells after transduction of T cells from different donors (*n* = 3 donors) with the duoCAR-encoding lentiviral vector (Fig. 2F), indicating there was minimal donor-to-donor variation in duoCAR expression, consistent with our previous findings (40). After duoCAR-T cells were incubated with TGF-β (10 ng/mL), they were either untreated or treated with the indicated HCW proteins (50 nM), and then SMAD2/3 phosphorylation was evaluated by flow cytometry. Treatment with HCW9218 and HCW9228, which incorporate functional TGF-βRII domains, inhibited TGF-β-induced phosphorylation of SMAD2/3 to levels comparable to duoCAR-T cells not treated with TGF-β (Fig. 2G and H). The results demonstrate that the TGF-βRII domains incorporated into the HCW9218 and HCW9228 effectively neutralized TGF-β, as indicated by the absence of downstream SMAD2/3 signaling. In contrast, cells incubated in the presence of TGF-β and HCW9238 or IL-15, both of which lack the TGF-βRII domain, displayed SMAD2/3 phosphorylation levels equivalent to duoCAR-T cells treated with TGF-β alone. Taken together, these data confirm the expected functional activities of the IL15SA and TGF-βRII domains linked by tissue factor in HCW9218.

### HCW9218 treatment stimulates expansion of CD8^+^ duoCAR-T cells

**Fig 3:**
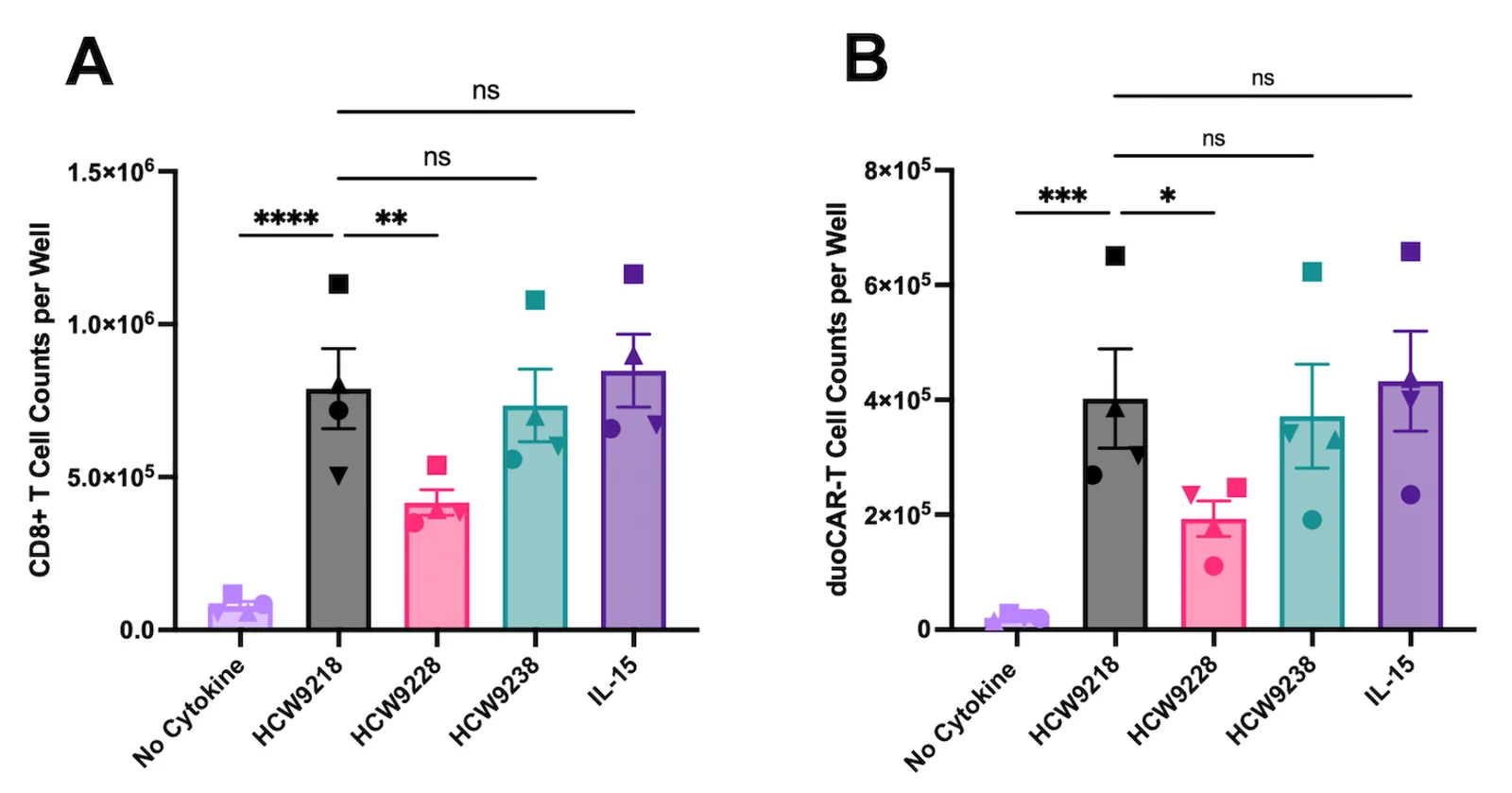
HCW9218 promotes IL-15-mediated expansion of primary CD8^+^ T cells and duoCAR-T cells in the presence of TGF-β. duoCAR-T cells were generated from four PWoH donors, each indicated by a unique marker shape. (**A**) The duoCAR-T cells were cultured with the indicated HCW proteins (50 nM), IL-15 (1 nM), or no cytokine for 5 days. Cell counts were determined by flow cytometry and gating on CD8^+^ T cells or (**B**) duoCAR-T cells. Data are presented as mean ± SEM of normalized values, averaged from technical triplicates across the four donors. Significance for panels A and B was determined by matched ordinary one-way ANOVA followed by Tukey’s multiple comparisons test. (*P < 0.05, **P < 0.01, ***P< 0.001, ****P < 0.0001).

We assessed whether the IL-15SA domain of HCW9218 promotes the proliferation of duoCAR-T cells. We transduced CD4^+^ and CD8^+^ T cells isolated from PBMCs obtained from people without HIV (PWoH) donors (*n* = 4 donors) with the duoCAR vector to generate duoCAR-T cells. After 3 days of culture in I10 (Iscove’s modified Dulbecco’s medium supplemented with 10% FBS, 1× GlutaMAX, 1× penicillin-streptomycin solution, HEPES buffer [100 mM]) with added IL-2 (100 U/mL), the cells were washed to remove IL-2 and were cultured in I10 containing no cytokine, IL-15 (1 nM), HCW9218, HCW9228, or HCW9238 (50 nM) for 5 days. HCW9218 treatment supported greater CD8^+^ T cell proliferation than the no-cytokine control or HCW9228, which contains an IL-15 domain mutated to reduce its functional activity (Fig. 3A). After treatment of the T cells with HCW9218, flow cytometric analysis gated on the CD8^+^ duoCAR+ subset demonstrated significantly greater expansion of the CD8 +duoCAR+ T-cell population compared with T cells treated with HCW9228 (Fig. 3B). These results indicate that the IL-15SA domain of HCW9218 supports the proliferation of both total CD8^+^ T cells and duoCAR-T cells.

### HCW9218 inhibits TGF-β-mediated suppression of duoCAR-T cell cytotoxic function

Multiple studies have established that TGF-β compromises T-cell effector and CAR-T cell function (47–50). Because PWH have elevated levels of TGF-β (19), it is likely that these elevated TGF-β levels would have an immunosuppressive effect that would mitigate the anti-HIV-1 activity of duoCAR-T cells. We therefore investigated whether TGF-β suppresses the anti-HIV-1 activity of duoCAR-T cells and whether HCW9218 treatment could counteract this inhibition. We evaluated the effect of TGF-β on the cytotoxic activity of duoCAR-T cells by incubating them with HEK293T cells, which express a gp120/41 fusion antigen on their surface and a luciferase reporter (HEK293T-gp120-Luc target cells). DuoCAR-T cells were co-cultured with HEK293T-gp120-Luc cells for 48 h in I10 at E:T ratios of 5:1 and 2:1 in the presence or absence of TGF-β (10 ng/mL or 20 ng/mL). The addition of TGF-β at either 10 ng/mL or 20 ng/mL equivalently suppressed duoCAR-T cell cytotoxicity (Fig. S3). Consequently, in subsequent experiments evaluating TGF-β suppression of anti-HIV-1 duoCAR-T cell activity, we used a concentration of 10 ng/mL, a concentration previously used in T-cell suppression studies (25, 47, 49).

**Fig 4:**
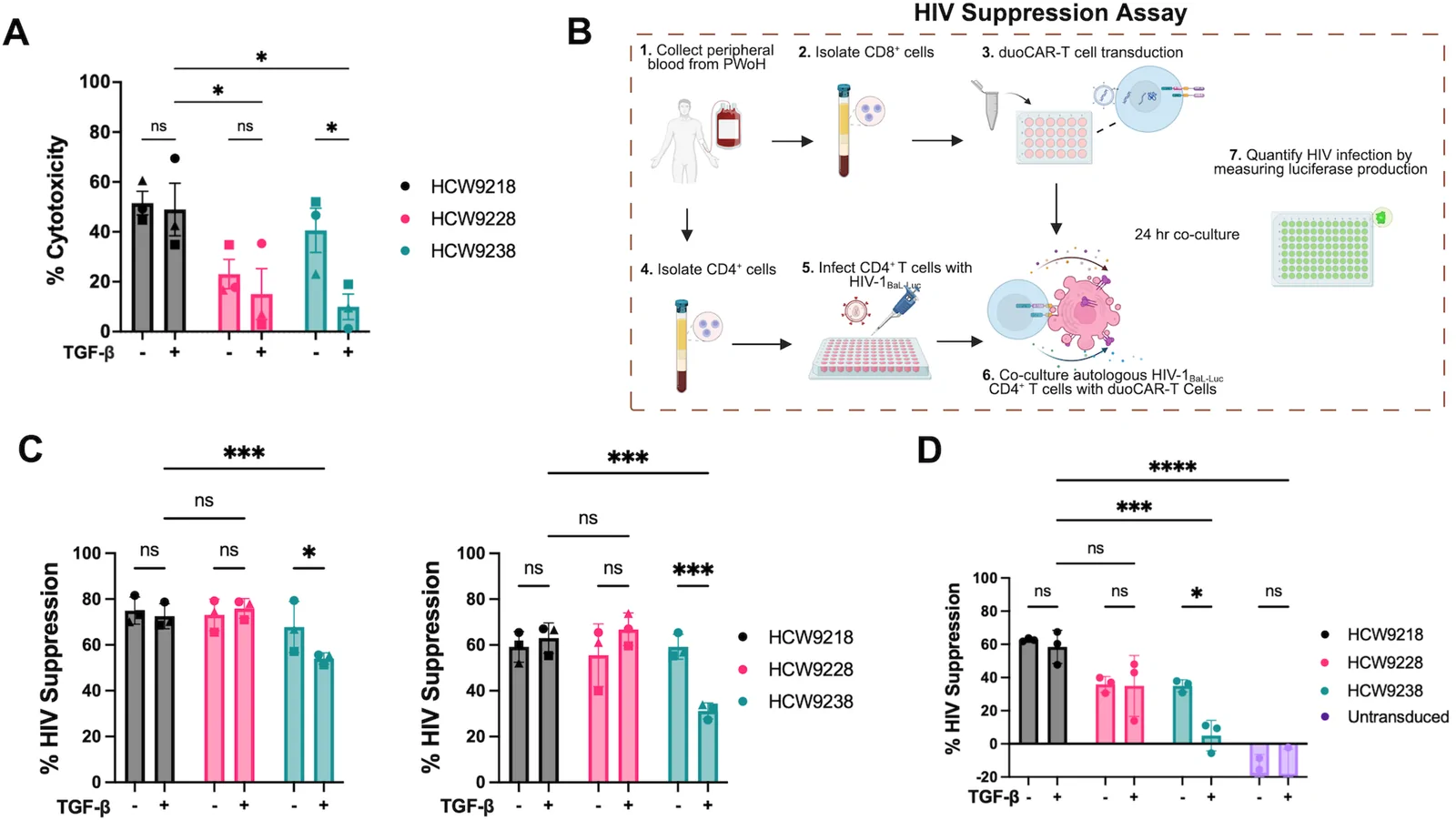
HCW9218 inhibits TGF-β-mediated suppression of duoCAR-T cell function generated from either PWoH or PLWH donors. (**A**) Luciferase cytotoxicity assay against HEK293T-gp120-Luc cells. The cytotoxic activity of the duoCAR-T cells was evaluated by quantifying their killing of HEK293T-Env+Luc+ (293T-gp120) cells which constitutively express gp120/gp41 fusion proteins on their membranes and firefly luciferase. CD4^+^ and CD8^+^ duoCAR-T cells were generated from PWoH and cultured at an E:T ratio of 2:1 with HEK293T-gp120-Luc cells for 48 h with and without added TGF-β (10 ng/mL). Cytotoxicity was measured by quantifying luciferase activity in the pelleted cells, a surrogate for cell number, enabling measurement of the reduction in 293T-gp120 cell number due to duoCAR-T cell-mediated lysis of the target cells. Data are presented as mean ± SEM of normalized values, averaged from technical triplicates across three donors. (**B**) Schematic detailing the protocol for the luciferase-based cytotoxicity assay using autologous HIV-1_BaL-Luc_-infected CD4^+^ T cells. (**C**) Autologous target CD4^+^ T cells infected with HIV-1_BaL-Luc_ were plated at 50,000 cells/well and co-cultured with duoCAR-T cells at an [E:T] of 1:40 (left panel) and 1:60 (right panel). The duoCAR-T cell number was adjusted while target cell number was held constant (1:40 = 1,250; 1:60 = 833 duoCAR-T cells). Cells were cultured with the indicated HCW proteins (50 nM) with and without added rhTGF-β (10 ng/mL) for 24 h. Cytotoxicity against HIV-1 infected target cells was calculated relative to matched HIV-1 infected target cell only controls. Data are presented as mean ± SEM of values normalized for % suppression of infection averaged from technical triplicates across three donors. (**D**) Luciferase-based cytotoxicity assay using cells from a PLWH donor. After CD8^+^ duoCAR-T cells were generated from a PLWH donor and pre-treated with the indicated HCW proteins with and without added rhTGF-β for 3 days, the duoCAR-T cells were cultured with autologous CD4^+^ T cells infected with HIV-1_BaL-Luc_ and the indicated HCW proteins (50 nM) with and without rhTGF-β (10 ng/mL) for 24 h at an [E:T] of 1:80. Cytotoxicity against HIV-1 infected target cells was calculated relative to matched HIV-1 infected target cell only controls. Negative values observed in cultures containing untransduced cells reflected higher luciferase activity than the corresponding HIV-1 infected target cell only control. Data are presented as mean ± SD of technical triplicates from one PLWH donor. Significance was determined by matched two-way ANOVA followed by Tukey’s multiple comparison test for panels A and C or by ordinary two-way ANOVA followed by Tukey’s multiple comparisons test for panel D. All statistical analysis was done in GraphPad Prism 10.4.0. (*P < 0.05, **P < 0.01, ***P < 0.001, ****P < 0.0001; ns = not significant).

We next examined the capacity of HCW9218 treatment to counteract TGF-β-mediated suppression of duoCAR-T cell cytotoxic function. DuoCAR-T cells were co-cultured with HEK293T-gp120-Luc cells with and without TGF-β (10 ng/mL) and the indicated HCW proteins. The presence of TGF-β significantly suppressed duoCAR-T cell cytotoxicity toward HEK293T-gp120-Luc cells (*P* < 0.05) in the presence of HCW9238, which lacks a functional TGF-β-neutralizing domain. In contrast, the presence of HCW9218 or HCW9228, both of which contain the TGF-β neutralization domain, blocked the capacity of TGF-β to inhibit duoCAR-T cell cytotoxicity (Fig. 4A). Notably, HCW9218, which retains IL-15 activity, conferred greater cytotoxicity than HCW9228, which has diminished IL-15 signaling (*P* < 0.05). These findings demonstrate that HCW9218 can enhance duoCAR-T-cell cytotoxicity through IL-15 stimulation while also inhibiting TGF-β-mediated suppression.

We next evaluated whether HCW9218 could prevent TGF-β-mediated impairment of duoCAR-T cell cytotoxicity against autologous CD4^+^ T cells infected with HIV-1_BaL-Luc_ as shown in the experimental protocol (Fig. 4B). After duoCAR-T cells were generated from three PWoH donors (Fig. S4), they were plated with a constant number of target HIV-1_BaL-Luc_-infected CD4^+^ T cells (50,000 cells/well) with either 1,250 or 833 duoCAR-T cells added to generate E:T ratios of 1:40 and 1:60, respectively (Fig. 4B). The cells were cultured with and without TGF-β and treated with either HCW9218, HCW9228, or HCW9238 and then co-cultured with autologous HIV-1_BaL-Luc_-infected CD4^+^ T cells. These E:T ratios were selected to model conditions of relatively low effector to target cell abundance, which may better reflect *in vivo* settings where effector cells are not present in excess. TGF-β did not significantly impair the cytotoxic activity of HCW9218- or HCW9228-treated duoCAR-T cells. In contrast, TGF-β treatment reduced cytotoxicity by HCW9238-treated duoCAR-T cells at the 1:40 (*P* < 0.05) and an even greater reduction at the 1:60 (*P* < 0.001) E:T ratios compared to HCW9238-treated duoCAR-T cells cultured in the absence of TGF-β (Fig. 4C). Additionally, HCW9218-treated duoCAR-T cells demonstrated significantly greater cytotoxic activity than HCW9238-treated duoCAR-T cells in the presence of TGF-β1 at both E:T ratios (*P* < 0.001). Collectively, these results indicate that HCW9218 prevents TGF-β-mediated inhibition of duoCAR-T cell cytotoxic function against HIV-1-infected target cells.

### HCW9218 inhibits TGF-β-mediated suppression of duoCAR-T cell cytotoxic function in PLWH

Treating PLWH with infused duoCAR-T cells to suppress the recurrence of viremia after the cessation of ART would utilize autologous duoCAR-T cells, which are derived from their own T cells. Because PLWH frequently exhibit elevated levels of TGF-β (19), it is important to determine whether TGF-β inhibits the anti-HIV-1 function of duoCAR-T cells derived from PLWH and whether HCW9218 binds TGF-β to neutralize its inhibitory effect on the anti-HIV-1 activity of duoCAR-T cells. For this purpose, we generated duoCAR-T cells from a PLWH donor as described above (Fig. S5) and evaluated their anti-HIV-1 activity by co-culturing them with autologous CD8-depleted PBMCs infected by an HIV-1 infectious molecular clone (IMC) expressing a *Renilla* luciferase reporter gene and the envelope from HIV_Bal_ (HIV-1-BaL-LucR) as previously described (39, 40). Infection with HIV-1-BaL-LucR enabled quantification of duoCAR-T cell-mediated cytotoxicity against autologous HIV-1-infected CD4^+^ T cells through measurement of luciferase activity in target cells. Baseline HIV-1 luciferase reporter activity in target cells following treatment with the HCW constructs alone is shown in (Fig. S6),

TGF-β reduced the cytotoxic activity of HCW9238-treated duoCAR-T cells, which possess IL-15 activity alone, whereas HCW9218- and HCW9228-treated duoCAR-T cells, both of which have the TGF-β-neutralizing domains, maintained their cytotoxic function in the presence of TGF-β (Fig. 4D). These findings demonstrate that HCW9218 preserves the antiviral activity of duoCAR-T cells generated from a PLWH donor, even under inhibitory conditions created by exogenously added TGF-β. A limitation in these results is that the study was performed in only a single PLWH donor due to our difficulty getting donors with minimal endogenous anti-HIV T cell activity to fully display anti-HIV duoCAR-T cell activity and future studies with additional PLWH donors will be important to confirm these results.

### Treatment with HCW9218 reactivates latent HIV-1-infected cells from an ART-suppressed PLWH donor

**Fig 5:**
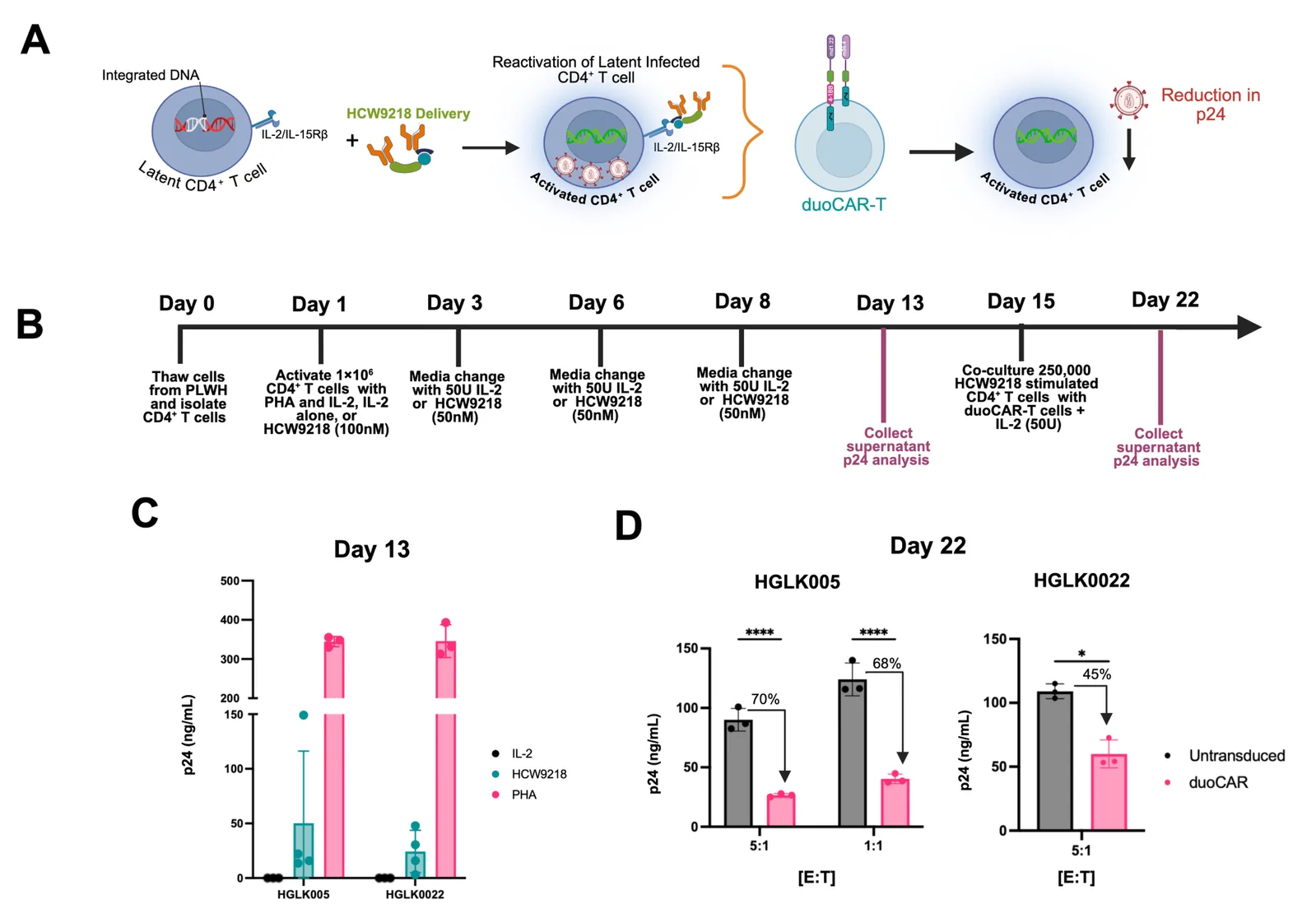
HCW9218-treated CD4^+^ T cells from PLWH produce HIV-1 p24, and autologous duoCAR-T cells suppress p24 antigen production in co-culture. (**A**) Schematic representation investigating HCW9218 reactivation of HIV-1 production by latent infected CD4^+^ T cells. (**B**) Experimental timeline. CD4^+^ T cells from ART-suppressed PLWH donors were cultured and treated with IL-2 alone, PHA + IL-2, or HCW9218. Supernatants were collected on day 13 for quantification of p24 antigen by ELISA. On day 15, HCW9218-treated CD4^+^ T cells (2.5 × 10^5^/well) were co-cultured with autologous untransduced CD8^+^ T cells or duoCAR T cells at the indicated (E:T) ratios in IL-2, and the culture supernatant was collected on day 22. (**C**) HIV p24 antigen production by PLWH CD4^+^ T cells cultured with HCW9218. Each dot represents one experimental replicate (individual culture well, averaged from triplicate ELISA plate measurements). Bars represent mean ± SD of 3 technical replicates for each donor. (**D**) Suppression of HIV-1 p24 antigen production by HCW9218-treated CD4^+^ T cells by autologous duoCAR-T cells. The numbers above the bars represent the percent reduction in p24 relative to untransduced controls at the same E:T ratio. Data are presented as mean ± SD of technical replicates (N=3 for IL-2 and PHA; *N*=4 for HCW9218). Significance for HGLK005 was determined using triplicate wells and two-way ANOVA followed by Sidak’s multiple comparisons test, and for HGLK0022 was performed using a paired parametric *t* test with Gaussian distribution was performed.

Treatment with IL-15SA can promote activation of latent HIV-1-infected CD4^+^ T cells and production of HIV-1, rendering them susceptible to elimination by HIV-1-specific cytotoxic T cells (38). Consequently, we evaluated whether the IL-15SA component of HCW9218 would activate latent HIV-1-infected CD4^+^ T cells to stimulate HIV-1 production, as measured by quantification of p24 antigen in the culture supernatant and thereby render these cells vulnerable to elimination by duoCAR-T cells (Fig. 5A and B). CD4^+^ T cells were isolated by immunomagnetic sorting from the PBMCs of two ART-treated PLWH with undetectable HIV-1 viremia (donors HGLK005 and HGLK0022) and cultured with added IL-2 (50 U/mL) with and without added PHA (4 µg/mL) or with added HCW9218 (50 nM) in the absence of ART. After 13 days, we quantified p24 antigen in the culture supernatant as an indicator of HIV production due to reactivation of latent HIV-1-infected cells and infection spread. As previously described (51, 52), while no p24 antigen was detected in the supernatant of the CD4^+^ T cells cultured with IL-2 from the ART-suppressed PLWH, p24 antigen production was markedly induced by PHA stimulation (Fig. 5C). Importantly, treatment with HCW9218 induced p24 antigen production, indicating its ability to reactivate latent HIV-1-infected CD4^+^ T cells from PLWH. Cell viability in these cultures ranged from 98%–99% across HCW9218-treated and PHA-treated cultures, indicating that the observed p24 production represented HIV production and not the release of p24 antigen by dying cells. The capacity of HCW9218 treatment to reactivate latent HIV infection in CD4^+^ T cells was further demonstrated with two additional ART-suppressed PLWH donors (Fig. S7). To further evaluate the capacity of the IL15SA domain of the HCW9218 to induce HIV production from latent HIV-1-infected T cells, we treated purified CD4^+^ T cells from an ART-suppressed PLWH donor with either HCW9218, added IL-2 alone, PHA with added IL-2, or the HDAC inhibitor vorinostat with added IL-2. Six days later, HIV-1 production was measured by quantifying HIV-1 RNA in the culture supernatant by RT-qPCR. HCW9218 treatment induced production of HIV-1 RNA at levels comparable to those induced by PHA, whereas HIV-1 RNA remained below the limit of detection in untreated and vorinostat-treated cultures (Fig. S8).

We next examined the capacity of autologous duoCAR-T cells generated from the PLWH donor T cells (Fig. S9) to suppress HIV-1 production following HCW9218-mediated reactivation of latent HIV-1-infected CD4 T cells. Fifteen days after the purified CD4^+^ T cells from the two PLWH were treated with HCW9218 and reactivated HIV-1 infection, they were either treated with autologous duoCAR-T cells or untransduced T cells, and HIV-1 production was evaluated by measuring p24 antigen release into the supernatant 9 days later. For donor HGLK005, p24 antigen supernatant levels were reduced by 70% or 68% after culture with duoCAR-T cells at E:T ratios of 5:1 or 1:1, respectively, and for donor HGLK022 by 45% after culture with duoCAR-T cells at an E:T ratio of 5:1 (Fig. 5D). Together, these findings suggest that HCW9218 can act as an LRA to induce p24 antigen release in CD4^+^ T cells from ART-suppressed PLWH donors and that autologous duoCAR-T cells can significantly reduce HIV-1 p24 production following HCW9218-mediated reactivation.

## DISCUSSION

ART suppresses HIV-1 replication in PLWH to below the limit of detection, yet reservoirs of latent HIV-1-infected cells persist and drive viral rebound when ART is interrupted, preventing an effective HIV-1 cure (53). This persistent reservoir of latent HIV-1-infected cells is a consequence of the inability of ART to eliminate latently infected cells (54), which has motivated investigators to focus on developing immune-based strategies capable of directly eliminating HIV-1-infected cells (55). CAR-T cells, such as duoCAR-T cells, represent an immune-based strategy for directly recognizing and eliminating HIV-1-infected cells. The duoCAR-T cells are engineered to express two binders designed to engage two conserved epitopes of gp120, reducing the likelihood of viral escape while protecting the duoCAR-T cells from HIV-1 infection (39, 40).

The optimal function of CAR-T cell-based therapies depends on the ability of circulating immune effector cells to maintain their anti-HIV-1 activity in the immunosuppressive environment found in many PLWH. One factor contributing to impaired immune responses in PLWH is the presence of elevated levels of TGF-β (15, 17, 19, 56). Multiple studies from the cancer field have reported that TGF-β impairs CAR-T cell proliferation, persistence, and cytotoxicity (57–59), and these suppressive effects on CAR-T cell function could be counteracted by TGF-β neutralization (49, 57, 60, 61). We postulated that the elevated TGF-β levels in many PLWH could similarly impair the function of anti-HIV-1 CAR-T cells, such as duoCAR-T cells.

In this study, we examined whether TGF-β inhibits the anti-HIV-1 activity of duoCAR-T cells and whether this inhibition can be overcome by treatment with HCW9218, a bifunctional fusion protein composed of two TGFβRII domains that bind and neutralize TGF-β, and an IL-15/IL-15Rα-sushi domain. In T cells, TGF-β signaling is primarily mediated through phosphorylation of SMAD2 and SMAD3, which regulate transcriptional programs; SMAD3 has been identified as a key signaling component activated by TGF-β to mediate its growth-inhibitory effects (62). We validated the functional activity of the TGF-βRII domains in HCW9218 by demonstrating their capacity to block exogenous TGF-β-induced SMAD2/3 phosphorylation in duoCAR-T cells, which was not affected by treatment with HCW9238, a control fusion protein with the IL-15SA domain but not with functional TGF-β-binding TGF-βRII domains (Fig. 2). We confirmed the biological function of the IL-15SA domain of HCW9218 by demonstrating its capacity to stimulate expansion of the IL-15-responsive cell line, M-07E, and proliferation of CD8+ T cells and duoCAR-T cells (Fig. 2 and 3). While HCW9238, a control fusion protein with a functional IL-15SA but non-functional TGF-β-binding domain, supported M-07E proliferation, it was unable to block TGF-β-mediated suppression of proliferation (Fig. S1). These results supported the potential use of HCW9218 treatment to both expand duoCAR-T cells and block the suppressive activity of TGF-β and thereby sustain duoCAR-T cell function in immunosuppressive environments with high levels of TGF-β, as reported for cancer-specific CAR-T cells (48, 57).

The TGF-β binding domain of HCW9218 preserved the functional activity of duoCAR-T cells in the presence of TGF-β. Despite the presence of TGF-β, duoCAR-T cells treated with HCW9218 retained their gp120-specific cytotoxicity against Env^+^ target 293T cells, while cultures treated with HCW9238, which lacks TGF-β neutralizing capacity, displayed a significant reduction in cytotoxicity (Fig. 4A). Similarly, in cytotoxicity assays against HIV-1 infected autologous CD4^+^ T cells, treatment with HCW9218, but not HCW9238, maintained the cytotoxic activity of duoCAR-T cells despite TGF-β treatment (Fig. 4C). Furthermore, HCW9218 treatment markedly inhibited TGF-β-mediated suppression of the antiviral function of duoCAR-T cells derived from a PLWH donor, whereas treatment with HCW9238, which lacks TGF-β neutralizing capacity, did not (Fig. 4D). These results support the capacity of TGF-β blockade to preserve the anti-HIV-1 activity of duoCAR-T cells derived from PLWH in a clinically meaningful context where elevated TGF-β levels may be present. Thus, HCW9218 may serve as an adjunct to maintain the proliferation, persistence, and cytotoxic function of duoCAR-T cells in PLWH, enhancing their ability to target and eliminate infected cells, including reactivated latent HIV-1-infected T cells.

Ultimately, achieving a functional cure for HIV-1 will not only require preserving the antiviral activity of effector cells but also eliminating latently infected CD4^+^ T cells that persist despite ART. HCW9218 contains an IL-15SA domain, which has been shown to activate and direct effector CD8+ T and NK cells to sites in lymph nodes and lymphoid tissues that may serve as sanctuary sites for latent HIV-1-infected cells not normally accessible to these effector cells (36, 37, 63). Furthermore, studies have demonstrated that treatment with an IL-15SA, N-803 (formerly ALT-803), can induce the *in vitro* reactivation of latent HIV-1-infected T cells (38, 52). While one study reported that treatment of SIV-infected macaques with ALT803 did not cause any consistent changes in plasma viral loads (37), other studies reported that N-803 treatment in combination with CD8^+^ T-cell depletion stimulated the *in vivo* reactivation of latent HIV-1-infected and SIV-infected cells as indicated by marked induction of plasma viremia in humanized mice and macaques, respectively (6, 64, 65). Of great interest was our observation that HCW9218 treatment of purified CD4^+^ T cells from ART-suppressed donors could promote HIV-1 production. The HIV-1 production from endogenous virus in the HCW9218-treated CD4^+^ T cells was significantly reduced by the addition of autologous duoCAR-T cells, indicating that duoCAR-T cells can suppress HIV-1 produced by reactivated latent HIV-infected T cells (Fig. 5). It is possible that treatment with IL-15 may increase the population of latent HIV-infected CD4^+^ T cells due to the capacity of IL-15 to induce proliferation of CD4^+^ T cells (66). However, expansion of the HIV reservoir was not observed in clinical trials which treated PLWH with the IL-15 superagonist N-803 (67, 68). One limitation of this study is that ART was not included in these cultures; therefore the measured p24 levels may reflect both initial induction of HIV-1 expression and spread of HIV-1 infection to other CD4^+^ T cells.

*In vivo* studies in nonhuman primates have shown that TGF-β neutralization can enhance immune activity and, in some settings, induce reactivation of HIV-1/SIV reservoirs (17, 18). While our study was not designed to investigate the contribution of TGF-β blockade on viral induction, the capacity of HCW9218 to block TGF-β may increase the LRA activity of its IL-15SA domain. Together, these results indicate that HCW9218 may provide a dual benefit: sustaining duoCAR-T cell anti-HIV-1 activity in high TGF-β environments while promoting HIV-1 expression that renders infected CD4^+^ T cells susceptible to clearance by concomitantly administered duoCAR-T cells. This dual activity could contribute to reducing the latent reservoir and increasing the capacity of duoCAR-T cells to provide a functional cure. To our knowledge, this represents the first demonstration that a bifunctional molecule combining IL-15 superagonist activity and TGF-β blockade can augment HIV-1-specific duoCAR-T cell therapy. Moreover, our findings demonstrate the *in vitro* capacity of duoCAR-T cells to suppress infection by endogenous HIV-1 generated by reactivation of latent HIV-infected T cells from ART-suppressed CD4^+^ T cells. Future studies are needed to evaluate whether the dual activity of HCW9218 can be harnessed in combination with duoCAR-T cells *in vivo* to reduce the HIV-1 reservoir. In conclusion, HCW9218 holds promise as an adjunct therapy to augment the efficacy of duoCAR-T cells in PLWH, particularly those with elevated TGF-β, and as a step toward sustained immune control and progress toward a functional cure.

## MATERIALS AND METHODS

### Lentiviral constructs

The duoCAR expression vector (MND-∆W) was generated as previously described (40). The bicistronic anti-HIV duoCAR encoding sequence (MND-∆W) was constructed by linking the coding sequences for each CAR domain (mD1.22 and m36.4) with coding sequences for the CD8 hinge, CD8 transmembrane domain, 4-1BB costimulatory domain, and/or the CD3ζ T-cell signaling domain. The md1.22 coding sequence was linked to the m36.4 coding sequence with a P2A sequence to enable equivalent expression of both CARs. The md1.22 CAR includes a 4-1BB and a CD3ζ signaling domain, and the m36.4 CAR includes a CD3ζ signaling domain without a co-stimulatory signaling domain (39).

### Isolation of PBMC

Leukapheresis to collect donor PBMCs was conducted using a consent and protocol approved by the Einstein Institutional Review Board (IRB). After leukapheresis, PBMCs were isolated by density centrifugation over Ficoll-Paque PLUS (Cytiva 45001750) solution in 50 mL conical tubes at 320 × *g* for 30 min. The PBMC layer at the interface was harvested, washed with PBS, and counted. The PBMCs were resuspended (50 × 10^6^ cells/mL) in FBS containing 10% dimethyl sulfoxide (DMSO), aliquoted, frozen, and stored at −150°C until use.

### Lentiviral vector production

Lentiviral vectors were produced as described previously (39) by transient transfection of HEK293T cells with four plasmids: a CAR transfer plasmid and three plasmids that encode VSV-G, HIV-1 Gag-Pol, or HIV-1 Rev. After transfection, the cell-free supernatant was collected 48 h later, and the vector was concentrated by centrifugation at 20,000 rpm for at least 18 h at 20°C. The viral titer was determined as the lowest concentration of viral supernatant capable of transducing Jurkat cells and calculated using the formula (cell number × percent positive × dilution factor) / (total volume).

### Infectious molecular clones of HIV-1 *Renilla* luciferase (LucR) reporter viruses

We used HIV-1 infectious molecular clones (IMC) that encode *Renilla* luciferase (LucR) reporter as a surrogate marker to quantify HIV-1 infection as described previously (40). HIV-1-LucR-IMC was generated by engineering replication-competent HIV-1 IMC using a pNL4-3 backbone to express, in *cis*, the LucR reporter gene and heterologous *env* sequences of selected HIV-1 strains as described (69). Virus stocks were produced by transfection of 293T cells with the indicated proviral plasmid DNAs, and the infectious titers (IU/mL) were determined on TZM-bl cells.

### Generation of anti-HIV-1 duoCAR-T cells from HIV-1 seronegative and seropositive donors

CD8^+^ and/or CD4^+^ T cells were purified from PBMCs by immunomagnetic sorting with human CD8^+^ and/or CD4^+^ microbeads (Miltenyi Biotec, Auburn, CA), as described (39, 40). The purified CD4^+^ and/or CD8^+^ T cells were plated in a 24-well plate and then activated by treatment with anti-CD3 (100 ng/mL) and anti-CD28 (2 μg/mL) for 2–3 days in I10 (Iscove’s modified Dulbecco’s medium supplemented with 10% FBS, 1× GlutaMAX [Ref# 35050-061, Gibco, Grand Island, NY], 1× penicillin-streptomycin solution [Ref# 30-002-CI, Corning Life Sciences, Tewksbury, MA], HEPES buffer [100 mM]) with added IL-2 (100 U/mL). After activation, the cells were plated in a 24-well plate (5 × 10^5^ cells) and were transduced with the duoCAR lentiviral vector (MOI of 40–50) in the presence of LentiBlast (1:10 dilution, OZ Biosciences, San Diego, CA, Ref# LBPX500), added to enhance transduction efficiency. After 3–4 days of culture, the transduced cells were washed with PBS, plated in a 24-well plate (1 × 10^6^ cells), and cultured for an additional 3–5 days with IL-2 (50 U/mL) added. The lentiviral vector transduction efficiency was confirmed by immunostaining and flow cytometry as described below.

### Cell growth assay

Nine days after transduction, duoCAR-T cells (5 × 10^5^ cells) were generated from four PWoH donors and were added to a 24-well plate and cultured in I10 media (1 mL). HCW9218, HCW9228, or HCW9238 proteins were added to the indicated wells (50 nM) or IL-15 (1 nM) or no cytokine, with or without added recombinant human TGF-β (10 ng/mL), (rhTGF-β1, Cat. No. 100-21, PeproTech, Cranbury, NJ), and cultured for 5 days. The total number of CD8^+^ T cells in each well was quantified by flow cytometry using an Aurora analyzer (Cytek Biosciences, Fremont, CA) and normalized based on the volume per cell culture well. Data were averaged from technical triplicates across the four donors.

### M-07E proliferation assay to evaluate IL-15 activity

The M-07E IL-15-responsive myeloid leukemia cell line (43) was grown in RPMI media with added FBS (20% vol/vol), penicillin (100 U/mL), streptomycin (0.1 mg/mL), and human GM-CSF (10 ng/mL) in 24-well plates. After establishing the culture, the M-07E cells were GM-CSF-starved for 3 days. The functional activity of the IL-15 domain in the HCW scaffolds was evaluated by adding them to the M-07E cells (1.5 × 10^5^ cells/well), culturing for 3 days and then quantifying proliferation using a sensitive colorimetric assay (Cell Counting Kit-8, Dojindo Laboratories, Gaithersburg, MD) and measuring absorbance at 450 nm. For dose-dependent proliferation, (1.5 ×10^5^ cells/well) were plated, and HCW9218, HCW9228, or HCW9238 (0 nM, 50 nM, 100 nM) was added to the cells in a 96-well plate and cultured for 3 days, followed by measuring absorbance at 450 nm.

### Evaluation of STAT5 phosphorylation of T cells by flow cytometry

CD4^+^ and CD8^+^ duoCAR-T cells generated from a person without HIV (PWoH) were cultured overnight in serum-free Iscove’s Modified Dulbecco’s Medium (IMDM) at 2 × 10⁶ cells/mL in a flat-bottom 96-well plate. Cells were left unstimulated or stimulated with IL-15, HCW9218, HCW9228, or HCW9238 for 30 min at 37°C. Cells were fixed with 1.6% paraformaldehyde for 10 min at room temperature, washed with FACS buffer, and permeabilized with chilled methanol for 30 min at 4°C. Following permeabilization, cells were washed and stained intracellularly with Alexa Fluor 647-conjugated anti-phospho-STAT5 (pSTAT5) antibody (BioLegend, Cat. No. 612599). After washing, cells were analyzed by flow cytometry.

### Cytotoxicity assay using HEK293T-Env+Luc+ target cells

The cytotoxic activity of the duoCAR-T cells was evaluated by quantifying their killing of HEK293T-Env+Luc+ (293T-gp120) cells, which we engineered to constitutively express gp120/gp41 fusion proteins on their membranes and a firefly luciferase gene to quantify cell numbers after duoCAR-T treatment by measuring luciferase levels (39). The 293T-gp120 cells (2 × 10^4^ cells/well) were seeded in 96-well plates and 30 min later, duoCAR-T cells were added to the wells at E:T ratios of 2:1 and 1:1 with the indicated HCW proteins (50 nM) with and without added TGF-β (10 ng/mL). After 2 days of culture, the plates were centrifuged at 1,300 rpm for 5 min, and the culture medium was carefully aspirated. Cells were lysed by adding of 1× lysis buffer (100 µL, Promega) to each well, and the plates were incubated for 90 min at room temperature in the dark and then centrifuged again at 1,300 rpm for 5 min. An aliquot of the supernatant (20 µL) was transferred to a 96-well plate, firefly luciferase substrate (100 µL, Promega) was added to each well, and bioluminescence was measured using a Berthold Automat Plus luminometer (Berthold Technologies, Oak Ridge, TN). Because firefly luciferase is constitutively expressed by the HEK293T-Env+Luc+ target cells, luminescence was proportional to the number of surviving target cells and inversely proportional to duoCAR-T cell-mediated cytotoxicity. The reduction in luciferase activity was used to calculate the percentage of cytotoxicity using the following formula:


$$\text{cytotoxicity}\;(\%)=\left(\frac{\text{luciferase of target cells alone}-\text{luciferase of experimental condition}}{\text{luciferase of target cells alone?}}\right)\times 100$$

### Assessment of duoCAR-T cell-mediated cytotoxicity against HIV-1-infected target cells derived from PWoH and PLWH

Autologous CD4^+^ T cells from donor PBMCs were purified by immunomagnetic sorting with anti-CD4^+^ magnetic beads (Miltenyi Biotec), activated by phytohemagglutinin A (PHA, 4 µg/mL), and cultured in complete I10 with added recombinant human IL-2 (100 U/mL). After 3 days, the cells were washed, plated in 96-well U-bottom plates (5 × 10^4^ cells/well), and infected by spinfection for 2 h with HIV-1-BaL-Luc (1 × 10^5^ infectious units) that encodes the *Renilla* luciferase (LucR) reporter as previously described (40). Autologous duoCAR-Ts were pre-treated with either HCW9218, HCW9228, or HCW9238 proteins (50 nM) in the presence or absence of TGF-β (10 ng/mL) for 3 days and added at an E:T of 1:40 and 1:60 to autologous HIV-1-infected CD4^+^ T cells. Effector-to-target (E:T) ratios were calculated based on total CD4^+^ T cells, recognizing that only a subset of the target cells are productively infected with HIV-1 *in vitro*. After 24 h of culture, HIV-1 infection was quantified by measuring luciferase levels using the Renilla Luciferase System (Promega) as previously described (39, 40). For studies using PLWH donors, we removed the confounding variable of endogenous anti-HIV-1 CTL activity by depleting CD8^+^ T cells from donor PBMCs using anti-CD8^+^ magnetic beads (Miltenyi Biotec) prior to infection.

### Evaluation of TGF-β-induced SMAD2/3 expression by flow cytometry

DuoCAR-T cells (1 × 10^5^ cells) were plated in 96-well flat-bottom plates and incubated overnight. The next day, the cells were pre-incubated with HCW9218, HCW9228, or HCW9238 proteins (50 nM) for 30 min at 37°C, followed by treatment with TGF-β (10 ng/mL) for 30 min at 37°C. The cells were treated with paraformaldehyde (2%) at room temperature for 15 min, washed with FACS buffer (150 
μL), and centrifuged for 4 min at 300 × g. The cells were incubated with ice-cold methanol (100 
μL) for 30 min at 4°C, washed once, and stained with a PE-conjugated mouse anti-pSMAD2/3 antibody (562586, BD Biosciences, San Jose, CA), washed again, resuspended in FACS buffer (200 
μL), and pSMAD2/3 expression was quantified by flow cytometry.

### HIV-1 p24 antigen ELISA

HIV-1 p24 antigen levels in culture supernatants were measured using HIV-1 p24 Antigen Capture Immunoassay Key Reagent Sets provided by the Biological Products Core of the AIDS and Cancer Virus Program (Frederick National Laboratory, NCI, NIH, Fort Detrick, MD). Standard curves were generated using p24 antigen standards provided, and sample concentrations were calculated by converting ELISA optical density values (OD) into concentrations (pg/mL) using a 4-parameter logistic (4PL) standard curve generated in GraphPad Prism (GraphPad Software, Boston, MA).

### Determination of latency-reversing activity of HCW9218

To evaluate whether HCW9218 can activate HIV-1 production from latent HIV-1-infected T cells and thereby function as a latency-reversing agent, we isolated CD4+ T cells from PLWH by immunomagnetic sorting and cultured the cells in I10 media (1 mL) in 24-well plates with either added HCW9218 (100 nM), PHA (4 μg/mL), and IL-2 (50 U/mL) or IL-2 (50 U/mL). PHA was washed from the wells on day 3, and fresh media containing IL-2 (50 U/mL) or HCW9218 (50 nM) was added on days 3, 6, and 8 and collected on day 13. For the 21-day stimulation, fresh media was added on days 4, 7, 10, and 15. On day 13, culture supernatants were collected, and p24 antigen concentrations were measured by ELISA as described above. After 15 days of culture, we harvested these cells and evaluated the capacity of autologous duoCAR-T cells, compared with untransduced T cells, to suppress HIV-1 infection initiated by reactivated latent HIV-1-infected T cells. We incubated cells from these cultures (2.5 × 10^5^ cells/well) with autologous duoCAR-T cells or untransduced CD8^+^ T cells with added IL-2 (50 U/mL) and co-cultured for 10 days. Supernatants were then collected for HIV-1 p24 antigen quantification by ELISA. The percent reduction was calculated using the following formula: percent reduction = 100 [1 − (p24 antigen concentration with added duoCAR-T cells/p24 antigen concentration with added untransduced T cells)].

### Quantification of HCW9218-induced HIV-1 production by RT-qPCR of cell culture supernatants

CD8^+^ T cells were depleted from peripheral blood mononuclear cells (PBMCs) by immunomagnetic sorting using human CD8 MicroBeads (Miltenyi Biotec, Auburn, CA). The CD8^+^ T cell-depleted PBMC fraction (flow-through) was collected, seeded (12 × 10⁶ cells/well) into 6-well plates in I10 medium, and the wells were treated with either HCW9218 (100 nM), vorinostat (330 nM), and IL-2 (50 U/mL), or PHA (2 μg/mL) and IL-2 (50 U/mL) for 6 days. HIV RNA in the culture supernatant was quantified using a modification of a previously described assay (70). Total RNA was extracted from cell culture supernatants (100 μL) using a QIAamp MinElute Virus Spin Kit (Qiagen, Germantown, MD) and reverse transcribed to generate cDNA using a high-capacity RNA-to-cDNA Kit (Applied Biosystems, Foster City, CA). RT-qPCR was performed using TaqMan Universal Master Mix II, no UNG (Applied Biosystems) with added cDNA (2 μL), and the HIV *gag* sequence-specific primers (5′-TCAGCCCAGAAGTAATACCCATGT-3′ [sense] and 5′-CACTGTGTTTAGCATGGTGTTT-3′) and an HIV *gag* sequence-specific (antisense) probe (6FAM-ATTATCAGAAGGAGCCACCCCACAAGA-TAMRA) as described previously (70). Cycle threshold values were calculated using a standard curve and samples with known copy numbers of absolute HIV DNA.

### Statistical analysis

Significance was determined by using the most appropriate analysis, either one-way ANOVA followed by Dunnett’s multiple comparison test or two-way ANOVA followed by Tukey’s (matched or unmatched) or Sidak’s multiple comparison test. All statistical analysis were performed using GraphPad Prism (version 10.4.0), with specific statistical tests indicated in each figure legend and the cutoff for statistical significance was a *P* < 0.05.

## Data Availability

Proprietary materials are available in limited quantities at a reasonable cost to the scientific community for noncommercial purposes, upon timely request under a Material Transfer Agreement (MTA) through HCW Biologics Inc. Fusion proteins generated in this study can be obtained from Dr. Niraj Shrestha (NirajShrestha@hcwbiologics.com) upon a timely request under an MTA.

## SUPPLEMENTAL MATERIAL

Supplemental material jvi.00051-26-s0001.docx Figures S1 to S9.

ASM does not own the copyrights to Supplemental Material that may be linked to, or accessed through, an article. The authors have granted ASM a non-exclusive, world-wide license to publish the Supplemental Material files. Please contact the corresponding author directly for reuse.
