# Supplementary material for: Enhancing HIV-1-specific CAR-T cell functional activity by treatment with a novel cytokine-based scaffold with IL-15 superagonist and TGF-β-neutralizing activity
Source: J Virol. 2026 Aug 14;100(9):e00051-26. doi: 10.1128/jvi.00051-26 (PMC13595942; doi:10.1128/jvi.00051-26)
Supplement: Supplementary file 1 [file jvi.00051-26-s0001.docx]

**Supplementary Figures**

**Figure S1. TGF-β Inhibition of M-07E Cell Proliferation Line is Prevented by Treatment with HCW9218**. M-07E cells were treated with the indicated HCW protein (50nM) or IL-15 (1nM) treatment with and without added TGF-β (10 ng/mL). After 3 days, cellular proliferation was measured using CCK-8 solution, and the level of 450nm absorbance was proportional to cell counts. Data are presented as mean ± SD of technical triplicates from one experiment. Significance was determined by ordinary two-way ANOVA followed by Tukey’s multiple comparisons test. (* = p<0.05, ** = p<0.01, *** = p<0.001, **** = p<0.0001).

**Figure S2. STAT5 signaling in duoCAR-T cells.** CD4⁺ and CD8⁺ duoCAR-T cells generated from a person without HIV (PWoH) were left unstimulated or stimulated with IL-15, HCW9218, HCW9228, or HCW9238 for 30 minutes. Cells were then fixed, permeabilized, and stained intracellularly with a phospho-STAT5 (pSTAT5) antibody for flow cytometric analysis. Representative flow cytometry plots are shown.

**Figure S3. TGF-β Treatment Suppresses the Cytotoxic Activity of duoCAR-T Cells**. CD4^+^/CD8^+^ duoCAR-T cells were generated from PWoH donor and cultured at an E:T of 5:1 or 1:1 with 293T-gp120-Luc for 2 days with and without added rhTGF-β (10ng/mL or 20ng/mL). Representative data are presented as the mean ± SD of technical triplicates from one donor. Significance was determined by ordinary two-way ANOVA followed by Tukey’s multiple comparisons test. (* = p<0.05, ** = p<0.01, *** = p<0.001, **** = p<0.0001

**Figure S4. PWoH DuoCAR-T Transduction Efficiency**. We quantified the duoCAR-T cell transduction efficiency in effector cells used in the HIV-1 suppression assays derived from PWoH donors (n=3 donors) by flow cytometric analysis. The first dot plot on the left for each donor (HGLK0086, HGK0123 and HGLK0122) displays the analysis of the untransduced duoCAR-T cells and the dot plots to its right display two (HGLK0086) or three (HGK0123 and HGLK0122) replicate analyses of the transduced duoCAR-T cells.

**Figure S5 DuoCAR-T Transduction Efficiency of T cells From a PWH Donor**. We used flow cytometric analysis to determine the duoCAR-T cell transduction efficiency for effector cells used in the HIV-1 suppression assay derived from a PWH donor. The first dot plot on the left for this donor (HGLK0072) displays the analysis of the untransduced duoCAR-T cells and the dot plot to its right displays analysis of the transduced duoCAR-T cells.

**Figure S6.** Baseline HIV-1 luciferase reporter activity in CD4⁺ T cells from a PLWH following treatment with HCW constructs, IL-15, or IL-2. Purified CD4⁺ T cells from a PLWH were infected with HIV-1_BaL_Luc for 3 days and subsequently treated with HCW9218, HCW9228, HCW9238, IL-15, or IL-2 in the presence of TGF-β (left) or absence of TGF-β (right) for an additional 3 days. Relative luminescence units (RLU) were measured in target cells that were not co-cultured with duoCAR-T cells or untransduced T cells, serving as the HIV-alone baseline control for the luciferase cytotoxicity assay. Each dot represents a technical replicate. Bars represent the mean ± SD.

**Figure S7**. **HIV-1 p24 antigen production by CD4⁺ T cells from PLWH**. Purified CD4⁺ T cells (1x10^6^ cells) from two PLWH donors were cultured in the presence of IL-2, HCW9218 or PHA and IL-2, and HIV-1 p24 levels in culture supernatants were measured after 21 days. Each symbol represents an individual technical replicate. Bars indicate the mean ± SD of technical replicates for each donor.

**Figure S8**. **HCW9218 induces HIV-1 RNA production by Day 6 after treatment.** CD8-depleted PBMCs from an ART-suppressed PLWH donor were cultured with IL-2 alone, HCW9218, PHA and IL-2, or vorinostat and IL-2. Extracellular HIV-1 RNA in culture supernatant was quantified by RT-qPCR at day 6 following stimulation. Values below the limit of detection were assigned a value of 0 for graphical representation. Data represents technical replicates for each condition from a single donor (HGLK005) and are presented as mean HIV RNA (copies/ml) with SD.

**Figure S9. DuoCAR-T Transduction Efficiency of T cells From a PLWH Donor**. Flow cytometric analysis was used to determine the duoCAR-T cell transduction efficiency for effector cells used to determine their capacity to suppress HIV-1 infection induced by reactivation of latent-HIV-1-infected CD4^+^ T cells by HCW9218 treatment. The first dot plot on the left for each donor (HGLK005 and HGK0022) displays the analysis of the untransduced duoCAR-T cells and the dot plots to its right display two replicate analyses of the transduced duoCAR-T cells.
